# Preparation of Novel Biodegradable Polymer Slow-Release Fertilizers to Improve Nutrient Release Performance and Soil Phosphorus Availability

**DOI:** 10.3390/polym15102242

**Published:** 2023-05-09

**Authors:** Yang Xiang, Yaqing Liu, Mingshan Gong, Yingfang Tong, Yuhan Liu, Guizhe Zhao, Jianming Yang

**Affiliations:** 1 Shanxi Province Key Laboratory of Functional Nanocomposites, College of Materials Science and Engineering, North University of China, Taiyuan 030051, China; zbdxxiangyang@163.com (Y.X.); gmsyzzll@163.com (M.G.); tongyyd@163.com (Y.T.); 19581540365@163.com (Y.L.); zgz@nuc.edu.cn (G.Z.); 2 Research Center for Engineering Technology of Polymeric Composites of Shanxi Province, North University of China, Taiyuan 030051, China; 3 School of Chemistry and Chemical Engineering, Anhui University of Technology, Ma’anshan 243032, China; 4 State Key Laboratory of Molecular Engineering of Polymers, Fudan University, Shanghai 200438, China

**Keywords:** biodegradable polymer, slow-release fertilizers, urea formaldehyde, nitrogen, available phosphorus

## Abstract

Inspired by the gradual collapse of carbon chain and the gradual release of organic elements into the external environment during the degradation of biodegradable polymers, a novel biodegradable polymer slow-release fertilizer containing nutrient nitrogen and phosphorus (PSNP) was prepared in this study. PSNP contains phosphate fragment and urea formaldehyde (UF) fragment, which are prepared by solution condensation reaction. Under the optimal process, the nitrogen (N) and P_2_O_5_ contents of PSNP were 22% and 20%, respectively. The expected molecular structure of PSNP was confirmed by SEM, FTIR, XRD, and TG. PSNP can release N and phosphorus (P) nutrients slowly under the action of microorganisms, and the cumulative release rates of N and P in 1 month were only 34.23% and 36.91%, respectively. More importantly, through soil incubation experiment and leaching experiment, it was found that UF fragments released in the degradation process of PSNP can strongly complex soil high-valence metal ions, thus inhibiting the phosphorus nutrient released by degradation to be fixed in the soil and ultimately effectively increasing the soil available P content. Compared with ammonium dihydrogen phosphate (ADP), a small molecule phosphate fertilizer that is easily soluble, the available P content of PSNP in the 20–30 cm soil layer is almost twice that of ADP. Our study provides a simple copolymerization method to prepare PSNP with excellent slow-release N and P nutrients, which can promote the development of sustainable agriculture.

## 1. Introduction

Phosphorus (P) is the main nutrient element necessary for plant growth [1]. Lack of P in soil will cause non-Biotic stress effect on plants and eventually lead to crop yield reduction [2]. Therefore, P supply is the main factor limiting crop yield. However, improper application of phosphate fertilizer can also have a certain negative impact on the ecological environment. Phosphate fertilizer mainly pollutes water bodies through erosion and runoff [3]. Water eutrophication caused by excessive P nutrients in surface water systems will lead to the reproduction of many aquatic plant and algae and damage the ecological balance of water bodies [4].

The utilization efficiency of plant P nutrients is often low when using traditional fast available P fertilizers, as phosphate is easily immobilized and inactivated by high-valence cations (Fe^3+^ and Al^3+^) in the soil [5]. The application of slow-release P fertilizer can continuously provide effective P nutrients to the soil for a certain period, thereby avoiding excessive P nutrients from being fixed by the soil, and effectively improving the P utilization efficiency of plants [6].

Up to now, many preparation methods for slow-release P fertilizers have been widely reported, among which coated slow-release P fertilizers are the most widely used [7,8]. Garcia et al. reported the use of pine lignin as a coating material for fast available P fertilizer to produce coated P fertilizer [9]. The application of coated P fertilizer improved the effectiveness of soil P and reduced soil fixation. Moreover, the coated P fertilizer prepared by encapsulating diammonium hydrogen phosphate with pine resin can maintain the effective P content of the soil at a high level for a long time [10,11]. Bernardo et al. have proved that layered bimetallic hydroxide hydrotalcite can well control the slow release of P nutrients after the absorption of phosphate, thus maintaining the soil available P content at a high level [12]. Another type is chemically synthesized polymer based slow-release P fertilizers, which are prepared by the dehydration and condensation reaction of phosphate with organic compounds containing hydroxyl groups (such as polyvinyl alcohol, starch, etc.) at high temperatures [13,14]. However, the matrix of these P fertilizers consisted mainly of carbon chain polymers, which had low content of available nutrients and high cost, so it is difficult to be really applied on a large scale.

Urea formaldehyde is the most widely used slow-release polymer nitrogen (N) fertilizer [15]. It can be slowly degraded and release N nutrients under the action of microorganisms [16]. Its N nutrient content is very high, usually more than 30%, and it is an efficient N fertilizer for improving crop yield and quality [17]. In addition, the urea formaldehyde molecular chain contains active groups (hydroxyl and amide groups, etc.), which provide the possibility for further modification [18]. Wang et al. used potassium dihydrogen phosphate and urea-formaldehyde prepolymers for copolymerization to prepare polymer fertilizer that can slow-release P and potassium nutrients [19]. However, the preparation process needed to be carried out at high temperature, which will result in high manufacturing cost, and high temperature reaction process may generate biuret harmful to the plant [20]. Therefore, it is necessary to further improve the preparation method and use a simple and environmentally friendly process to prepare polymer fertilizer that can release N and P slowly.

In this study, we use urea phosphate as the reaction material and condense it with urea formaldehyde prepolymer to prepare a new type of biodegradable polymer slow-release fertilizer containing nutrient N and P (PSNP). The fertilizer has excellent N and P nutrient slow-release performance and is a new type of biodegradable polymer fertilizer for improving soil long-term fertility.

## 2. Materials and Methods

### 2.1. Materials

Formaldehyde (37 wt.%), urea, potassium hydroxide (KOH), ammonium dihydrogen phosphate (ADP), and phosphoric acid (H_3_PO_4_) are purchased from Damao factory, Tianjin, China. All chemical reagents are analytical grade and used without further purification.

### 2.2. Methods

#### 2.2.1. Preparation of Urea Phosphate

The prepared H_3_PO_4_ solution with a mass fraction of 55% was added to a three necked flask, and the temperature was raised to 60 °C. urea with a mass ratio of 1:1 to H_3_PO_4_ was added. The clarified solution was obtained after reaction for 30 min. Frozen and crystallized the solution overnight in a refrigerator to obtain urea phosphate crystals. Dried the crystal in an oven to constant weight to obtain the final product urea phosphate.

#### 2.2.2. Preparation of PSNP

25 mL of formaldehyde and 16 g of urea were added to the reaction vessel, and the pH of the system was adjusted to 8 using 5% KOH. The system temperature was controlled at 40 °C for 120 min. Then, 16 g of prepared urea phosphate crystals were added to the reaction system, and the reaction temperature was controlled at 40 °C. The white viscous product was obtained after 60 min of reaction. The product was granulated and dried to obtain a novel biodegradable polymer slow-release fertilizers PSNP. It should be noted that the granulation process of PSNP was consistent with the previous method of our group [18].

For comparative analysis, urea formaldehyde fertilizer (UF) was prepared using the same ratio of urea and formaldehyde under the same process conditions, without the addition of urea phosphate. The nutrient content of UF and NP is shown in Table 1.

#### 2.2.3. SEM Analysis

The surface morphologies of samples were observed using a scanning electron microscopy (SEM, Hitachi SU8010, Tokyo, Japan).

#### 2.2.4. FTIR Analysis

The functional groups and chemical construction of fertilizers were investigated using a Fourier-transform infrared spectrometer (FT-IR, Nicolet iS50, Waltham, MA, USA). The sample was ground into powder and tested directly in reflection mode. The wavenumber was in the range of 4000–500 cm^−1^, the resolution was 8 cm^−1^, and the accumulated scanning times were 16.

#### 2.2.5. XRD Analysis

X-ray diffractometry (XRD, HAOYUAN DX-2700B, Dandong, China) was used to identify the crystal structures of samples in the 2θ range of 5–80°. The scanning rate at room temperature is 0.03°/s. The crystallinity was calculated by MDI Jade software (Version 6.5).

#### 2.2.6. Thermal Analysis

Thermogravimetric analyzer (TG, TA Q50, New York, NY, USA) was used to test the thermal properties of materials. The test conditions were as follows: under N atmosphere, the temperature range was 40–800 °C, and the heating rate was 20 °C/min.

#### 2.2.7. Slow-Release Performance in Soil

First, 1 g of PSNP was mixed with 200 g of dry soil sieved through a 40-mesh sieve and placed in a 250 mL plastic bottle. The soil used was collected from an uncontaminated crop farming area in Taiyuan City; the basic physical and chemical properties were shown in Table 2. At room temperature, the water content of the soil in the plastic bottle was controlled at about 30%. After 1, 3, 5, 7, 10, 15, 20, 25, and 30 days, the remaining fertilizer particles were carefully separated and removed from the soil, weighed, and analyzed further. They were dried to constant weight at 80 °C. The residual fertilizer samples were digested by H_2_SO_4_-H_2_O_2_ digestion method, and the contents of N and P in the digestion solution were determined using a Kjeldahl N analyzer (HN-01, Shanghai Yonggui Analytical Instrument Co., Ltd., Shanghai, China) and an ultraviolet spectrophotometer (UV759S, Shanghai Precision Scientific Instrument Co., Ltd., Shanghai, China), respectively.

#### 2.2.8. Effect on Soil Available P

0.1 g of PSNP and ADP with the same amount of P milled through an 80-mesh sieve were mixed evenly with 500 g air-dried soil, respectively, and packed into a 500 mL plastic bottle. The soil moisture was about 30% to simulate soil moisture. A blank control without fertilizer (CK) was also set up. The soil available P content was measured after 90 days of incubation. The brief measurement method: the soil was extracted with 0.5 mol/L NaHCO_3_ at pH 8.5, and the available P content is determined by molybdate colorimetry.

#### 2.2.9. Vertical Migration Effect of Soil Available P

**Figure 1:**
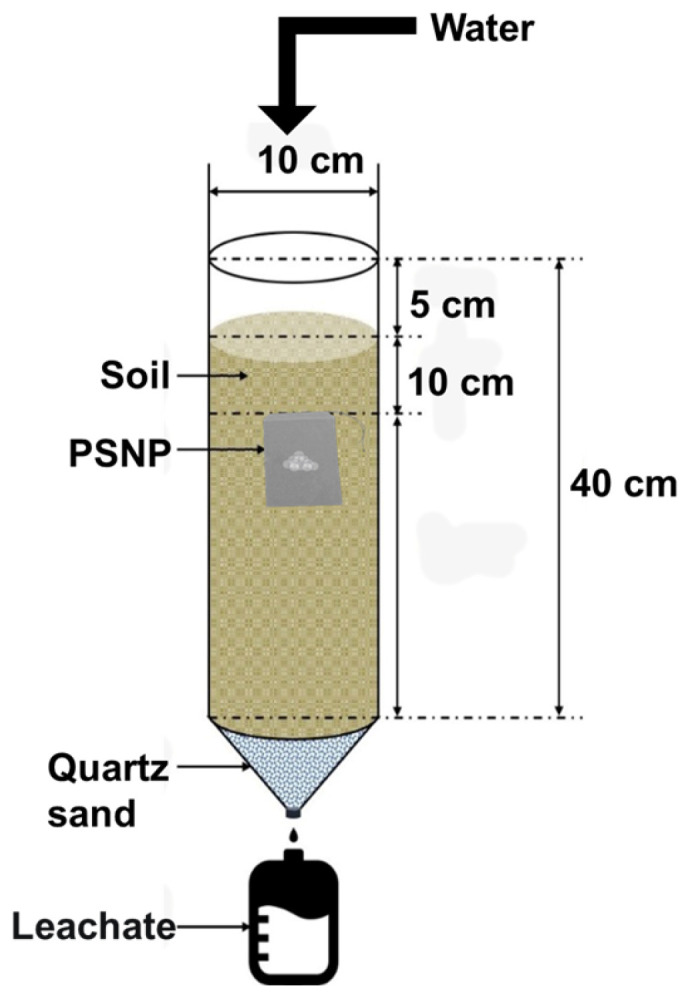
Schematic diagram of the device for soil available P migration experiment.

The device diagram for soil available P migration was shown in Figure 1. Loaded the air-dried soil into the soil column tube (height 40 cm, diameter 10 cm). Before loading the soil, lay a layer of clean quartz sand (diameter of 2~3 mm) washed with deionized water and dried at the bottom of the soil column first, then loaded the soil to 15 cm away from the top of the soil column, and then put in 0.5 g fertilizer packed with 200-mesh nylon net bag. The treatment was as follows: PSNP and ADP with the same amount of P were added, and a blank treatment without fertilizer (CK) was also set up. The soil continued to be loaded to a position 5 cm away from the top of the soil column to form a simulated soil column about 35 cm high. A 500 mL beaker was placed at the bottom of the soil column to collect leachate. Deionized water was added from the top (a small amount of about 10 mL at a time) until the leachate flowed out at the bottom, so that the soil in the soil column was fully moistened. Then, 200 mL of deionized water was added every 15 days (a small amount of about 10 mL at a time). After 90 days of incubation, the average available P content of the soil column at different depths (0–10 cm, 10–20 cm, 20–30 cm) was measured, and the migration behavior of soil available P was evaluated according to the data.

#### 2.2.10. Data Analyses

Origin 9.0 software (Origin Lab, Northampton, MA, USA) was used to draw curves. For each statistical analysis, unless otherwise specified, data in triplicate shall be used for each material processing and each sampling cycle. The statistical significance of the difference between samples was determined by Duncan multirange test, and *p* < 0.05 is the significant level.

## 3. Results and Discussion

### 3.1. Synthesis Mechanism

**Figure 2:**
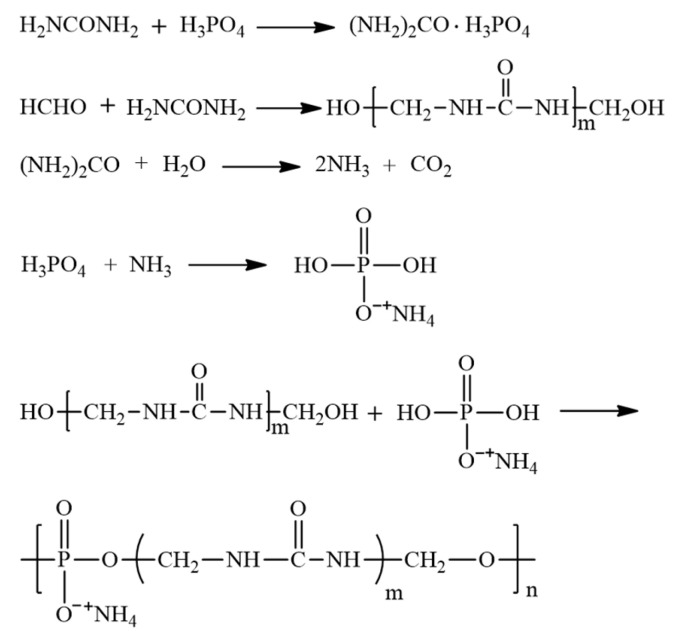
Synthesis Mechanism of PSNP.

The synthesis mechanism of PSNP was shown in Figure 2. After the H_3_PO_4_ and urea were fully mixed, the lone pair electrons on the N atom in the urea molecule would combine with the hydrogen ions ionized by the phosphoric acid to form a coordination bond, thereby forming a phosphate urea crystal. Under high temperature conditions caused by heat of reaction, some of urea could decompose and release ammonia and carbon dioxide, which in turn would react with H_3_PO_4_ to produce ammonium phosphate. At this time, the urea formaldehyde oligomer generated by the reaction of formaldehyde and urea would undergo a condensation reaction with ammonium phosphate, ultimately resulting in the successful synthesis of PSNP.

### 3.2. Structural Characterization

#### 3.2.1. SEM Analysis

**Figure 3:**
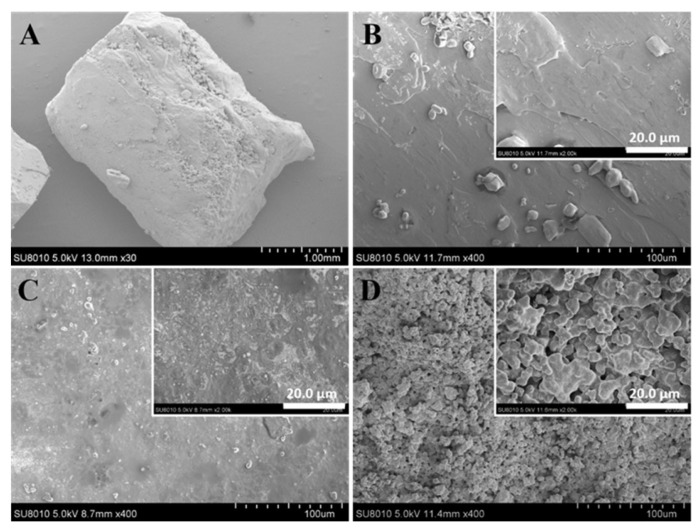
SEM images of urea phosphate (**A**,**B**), UF (**C**), and PSNP (**D**).

The microstructure of urea phosphate, UF, and PSNP was shown in Figure 3. From Figure 3A, urea phosphate was a prismatic crystal, stacked together by parallel layered structures, and bound together by hydrogen bonds between layers, belonging to a rhombic crystal [21]. From the locally enlarged view of urea phosphate (Figure 3B), its crystal surface was relatively flat. From Figure 3C, the surface of UF was relatively smooth and there was no crystal structure on the surface of traditional urea formaldehyde fertilizer, indicating that the polymerization reaction between urea and formaldehyde was relatively complete and the polymerization degree was high [22]. From Figure 3D, the overall structure of PSNP is composed of many irregular micrometer sized blocks, mainly because urea phosphate was a strong acid salt. During the preparation of PSNP, it could strongly catalyze the condensation reaction between urea and formaldehyde, and the macromolecules formed by condensation quickly separated from the solution and were assembled into irregularly shaped particles. In addition, ammonia and carbon dioxide released by decomposition of urea, which will also contribute to the formation of pore structures on the surface of PSNP particles. In addition, these porous structures increase the contact area with soil microorganisms, further contributing to the sustained and stable release of fertilizer nutrients.

#### 3.2.2. FTIR Analysis

**Figure 4:**
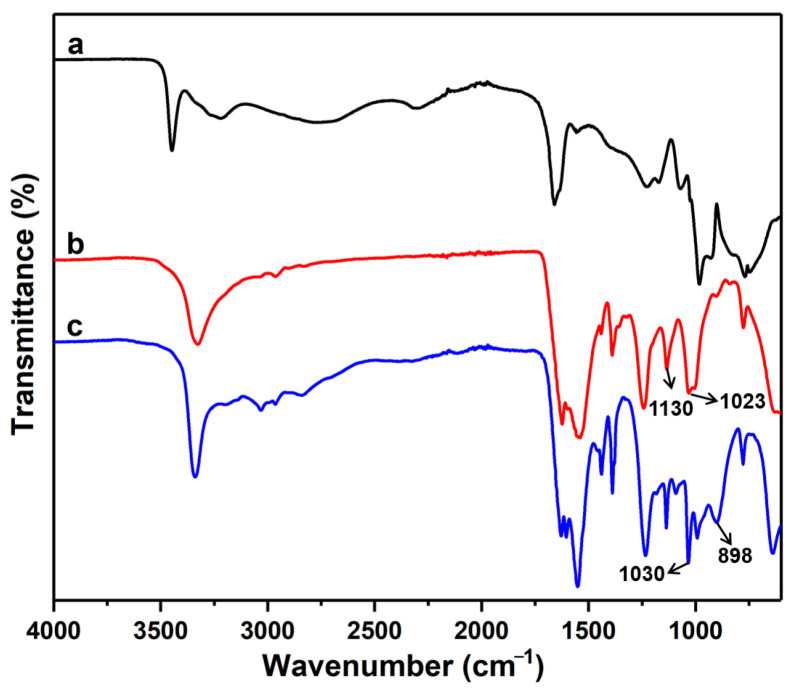
FTIR spectra of urea phosphate (a), UF (b), and PSNP (c).

The FTIR spectra of urea phosphate, UF, and PSNP were shown in Figure 4. Curve a in Figure 4 showed that the FTIR spectrum of urea phosphate was consistent with the peak position reported by Abou Okeil et al., indicating the successful preparation of urea phosphate [23]. Curve b in Figure 4 showed the FTIR spectrum of UF, where the characteristic absorption peak at 1130 cm^−1^ could be attributed to the N-CH_2_-N asymmetric stretching vibration absorption peak in UF, while the characteristic absorption peak at 1023 cm^−1^ could be attributed to the C–O stretching vibration absorption peak in—CH_2_OH in UF. It could be observed that the ratio of characteristic absorption peak intensities of UF at 1023 cm^−1^ and 1130 cm^−1^, A1023/A1130, was significantly greater than the FTIR of UF prepared by Xiang et al. [24]. This indicated that at a higher ratio of formaldehyde to urea, the UF obtained had more terminated hydroxyl groups, which was very conducive to the esterification reaction between urea-formaldehyde oligomers and phosphates. As shown in curve c in Figure 4, the characteristic absorption peaks of PSNP at wavenumbers 1030 cm^−1^ and 898 cm^−1^ could be attributed to P-O bond symmetric and asymmetric stretching vibration absorption peaks, respectively, indicating the presence of many phosphate ester bonds in the PSNP molecule, confirming that PSNP indeed had the structure shown in Figure 2. On the other hand, by comparing curves b and c in Figure 4, PSNP did not exhibit a corresponding C-O stretching vibration absorption peak in—CH_2_OH at a wavenumber of 1023 cm^−1^, indicating a higher reaction degree between terminal hydroxyl groups of urea formaldehyde oligomers and phosphate, indirectly reflecting a higher yield of PSNP.

#### 3.2.3. XRD Analysis

**Figure 5:**
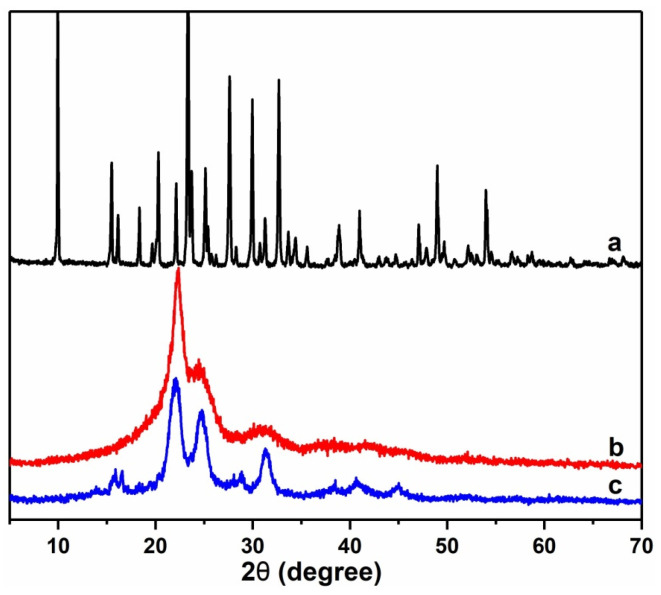
XRD patterns of urea phosphate (a), UF (b), and PSNP (c).

XRD can be used to study the crystal structure of the sample. From Figure 5a, the X-ray diffraction peak of the phosphate urea prepared in this study was consistent with the peak position on the JCPDS phosphate urea standard spectrum, indicating that the phosphate urea had indeed been successfully prepared. As shown in Figure 5b, there was a clear crystallization peak at an angle of 22.4°, indicating that UF was a semicrystalline resin, which was consistent with literature reports [25]. As shown in curve c in Figure 5, compared to UF, PSNP exhibited new characteristic absorption peaks at 15.89°, 16.55°, and 24.74°, indicating the existence of new crystal forms in PSNP. These crystallization peaks match the crystallization peaks of ammonium polyphosphate, indicating the presence of a small amount of ammonium polyphosphate in PSNP. On the other hand, by comparing curves a and c in Figure 5, there was no longer a crystallization characteristic peak of urea phosphate in PSNP, which clearly indicated that urea phosphate was basically completely reacted by the subsequent condensation reaction during the preparation of PSNP, with a high degree of reaction.

#### 3.2.4. Thermal Analysis

**Figure 6:**
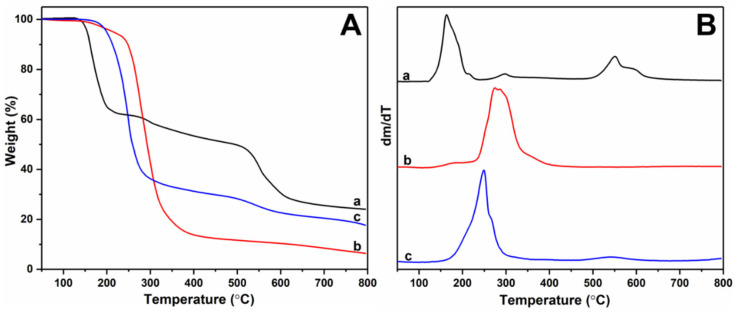
TG curves (**A**) and DTG curves (**B**) of urea phosphate (a), UF (b), and PSNP (c).

The TG/DTG curves of urea phosphate, UF, and PSNP were shown in Figure 6. The thermal degradation process of urea phosphate could be divided into three stages, namely 127–231 °C, 261–321 °C, and 490–658 °C, with corresponding thermal weight losses of 37.82%, 4.07%, and 23.55%, respectively. This indicated that the initial decomposition temperature of urea phosphate was very low, and its thermal stability was not high [26]. During this stage, urea phosphate would decompose and release ammonia gas. Afterwards, the two thermal decomposition processes of urea phosphate at 261 °C and 490 °C were consistent with the thermal decomposition of ammonium polyphosphate, indicating that some urea phosphate would be converted into ammonium polyphosphate at high temperatures [27]. For UF, its maximum thermal weight loss occurred in the temperature range of 228–361 °C, with a thermal weight loss of 76.50%, which was consistent with the results of UF thermal stability testing by Xiang [18]. On the other hand, it also indicated that the different ratios of formaldehyde and urea do not result in significant differences in the thermal stability of the final UF. For PSNP, its thermal stability was significantly lower than that of UF, mainly due to the decrease in the regularity of molecular chain arrangement after the polymerization of phosphate ester groups into the urea formaldehyde molecular chain, making it easier to decompose at lower temperatures. Comparing the curves a and c in Figure 6B, PSNP also exhibited a significant thermogravimetric peak at 479–622 °C, indicating that PSNP contained a small amount of ammonium polyphosphate. Therefore, according to Figure 2, not only did the intermediate ammonium dihydrogen phosphate undergo condensation reaction with urea formaldehyde oligomer to generate PSNP, but also a small amount condensed with urea to generate ammonium polyphosphate. This result was consistent with the above XRD analysis. The thermal analysis results also indirectly confirmed that PSNP was indeed successfully prepared.

### 3.3. Slow-Release Behavior of PSNP in Soil

**Figure 7:**
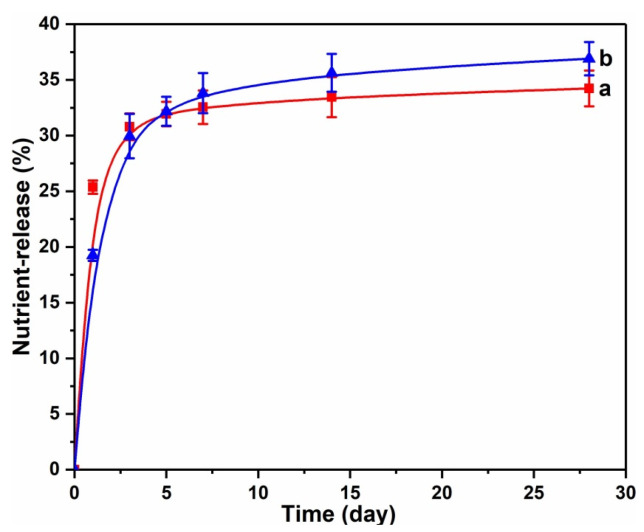
Slow-release profiles of N (a) and P (b) from PSNP in soil.

Urea phosphate is easily soluble in water, and its N and P nutrients are both available nutrients [28]. From Figure 7, the release rate of N and P nutrients in the PSNP prepared by the condensation of urea phosphate and urea formaldehyde oligomer is relatively slow in the soil, indicating that it is a slow-available nutrient. In 0~5 days, the slow-release rate of N and P in PSNP was faster. This was because PSNP contains a certain number of small molecules and oligomers, which were more easily soluble in water. Therefore, after being applied to the soil, the soluble components in PSNP first dissolved in the soil solution and quickly released to the outside. After 5 days of degradation, the release of N and P nutrients in PSNP tended to flatten out. By the 30th day, the cumulative release rates of N and P were 34.23% and 36.91%, respectively. This indicated that the slow release of P nutrients will be greatly improved after phosphate polymerization into the urea formaldehyde molecular chain, which will greatly help to improve the P nutrient utilization efficiency of fertilizers. The molecular structure of PSNP was basically the same as that of fertilizer (PSRF/KCl) prepared by Li et al., the nitrogen release rate of PSNP was significantly higher than that of PSRF/KCl (less than 5%) [15]. However, in general, plants have a great demand for nitrogen during the whole growth period, so PSNP has better slow-release performance for N and P in soil.

### 3.4. Effect on Soil Available P Content

**Figure 8:**
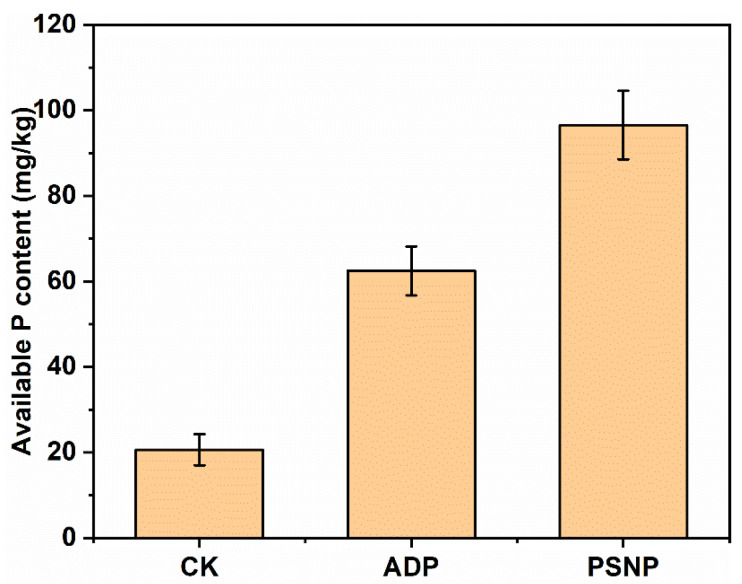
Soil available P content after 90 d of incubation.

The effects of each treatment on soil available P are shown in Figure 8. The content of available P in soil fertilized with ADP and PSNP was 62.47 mg/kg and 96.54 mg/kg, respectively. The results showed that PSNP fertilizer could promote soil available P content. Because P in PSNP fertilizer has a good slow-release effect, its slow-release process can reduce the fixed amount of metal cations in the soil. In addition, the UF segment in PSNP would be broken in the degradation process to form many medium or small molecular fragments, namely UF fragments, which will form stable complexes with high-valence metal ions, and the complexes can reduce P fixation and facilitate P migration in soil and uptake by crops [29]. Obviously, the use of PSNP is a feasible method to improve P nutrition in agricultural soil.

### 3.5. Vertical Migration Effect of Soil Available P

**Figure 9:**
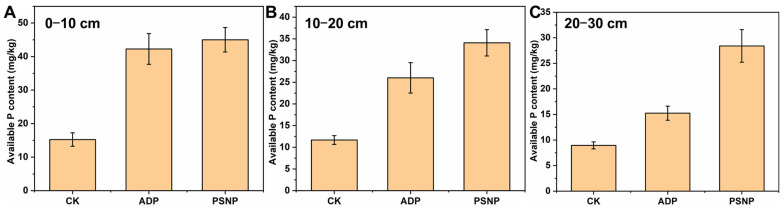
Soil available P level of different vertical migration distances: (**A**) (0–10 cm), (**B**) (10–20 cm), (**C**) (20–30 cm).

The vertical distribution of soil available P is a key index to measure crop growth and evaluate the effectiveness of P fertilizer [30]. The available P levels of soil at different depths can be determined by soil column leaching experiments [31]. As shown in Figure 9, there was no significant difference between the available P content of PSNP and that of ADP in soil at a depth of 0 to 10 cm. the available P content of PSNP was significantly higher than that of ADP in soil at a depth of 10 to 20 cm. The available P content of PSNP was almost twice that of ADP in soil at a depth of 20 to 30 cm. The results showed that the slow release of P by PSNP macromolecular chains and the complexation between UF fragments released by degradation and high-valence metal ions could greatly improve the migration ability of P in soil and promote the entry of available P into cultivated layer with irrigation water, and it could finally be absorbed and utilized by plants.

### 3.6. Mechanism of Improving P Utilization Efficiency

**Figure 10:**
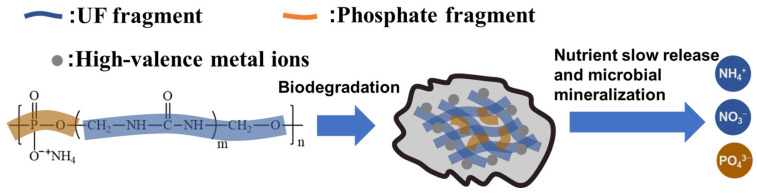
Mechanism of improving P utilization efficiency by PSNP.

Combined with the above analysis, the possible mechanism of improving P utilization efficiency by PSNP can be obtained, as shown in Figure 10. After the application of PSNP in soil, under the action of soil microorganisms or water molecules, the PSNP will degrade and first form a complex containing UF fragments and phosphate fragments, in which the amide group in the UF fragments will be complexed with high-valence metal ions to form stable metal complexes, thus reducing the concentration of metal ions in the environment around the phosphate fragments. Then, the phosphate fragments gradually degrade to form phosphate ions that can be absorbed by plants and finally provide stable soil available P content to be absorbed and utilized by plants. Therefore, the copolymerization of phosphate and UF oligomer is an efficient means to improve the P utilization rate of fertilizer.

## 4. Conclusions

In this study, urea phosphate was used as the reaction material and condensed with urea formaldehyde prepolymer to prepare a new type of biodegradable polymer slow-release fertilizer containing nutrient N and P. The fertilizer had excellent slow-release performance for N and P nutrients and gave P nutrients excellent migration characteristics.

The content of N and P (P_2_O_5_) in PSNP was 22% and 20%, respectively. After co-polymerization of phosphate and urea formaldehyde prepolymer, the nutrient release performance of the fertilizer can be significantly improved. The cumulative release rates of N and P in soil for one month were only 34.23% and 36.91%, respectively.

Use of PSNP significantly promoted the vertical migration ability of P nutrients and enhanced the available P level in soil. The migration data showed that the soil available P content under PSNP treatment was about twice that under ADP treatment in the 20–30 cm soil layer.

Polymer fertilizers with excellent slow-release performance for N and P nutrients can be obtained by conventional polycondensation reactions. The production process was simple and suitable for industrial production.

## Figures and Tables

**Table 1:** Characteristics of the slow-release fertilizers.

Characteristics	UF	PSNP
N content (%)	29	22
P (P_2_O_5_) content (%)	\	20

**Table 2:** Basic physical and chemical properties of the soil used in this study.

Soil Properties	pH (1:2.5, Soil:Water)	Cation Exchange Capacity (cmol/kg)	Organic Matter (g/kg)	Total N (g/kg)	Available P (mg/kg)	Available K (mg/kg)
Value	7.91	15.20	18.01	0.92	24.08	55.14

## Data Availability

Not applicable.
